# Infectivity of *Giardia duodenalis* Cysts from UV Light-Disinfected Wastewater Effluent Using a Nude BALB/c Mouse Model

**DOI:** 10.5402/2013/713958

**Published:** 2013-01-14

**Authors:** Luciana Urbano dos Santos, Delma Pegolo Alves, Ana Maria Aparecida Guaraldo, Romeu Cantusio Neto, Mauricio Durigan, Regina Maura Bueno Franco

**Affiliations:** ^1^Laboratory of Oxidation Processes, Department of Sanitation and Environment, School of Civil Engineering, Architecture and Urbanism, University of Campinas (UNICAMP), C.P. Box 6021, 13083-852 Campinas, SP, Brazil; ^2^CEMIB Multidisciplinary Centre for Biological Investigation, University of Campinas (UNICAMP), C.P. Box 6095, 13083-877 Campinas, SP, Brazil; ^3^Laboratory of Helminthology, Department of Parasitology, Institute of Biology, University of Campinas (UNICAMP), C.P. Box 6109, 13083-970 Campinas, SP, Brazil; ^4^Laboratory of Microbiology, Society for Water Supply and Sanitation (SANASA), Street Abolição 2.375, 13045-750 Campinas, SP, Brazil; ^5^Laboratory of Genetic and Molecular Analysis, Center of Molecular Biology and Genetic Engineering (CBMEG), Institute of Biology, University of Campinas (UNICAMP), C.P. Box 6109, 13083-875 Campinas, SP, Brazil; ^6^Laboratory of Protozoology, Department of Animal Biology, Institute of Biology, University of Campinas (UNICAMP), C.P. Box 6109, 13083-970 Campinas, SP, Brazil

## Abstract

*Giardia duodenalis* is a protozoan of public health interest that causes gastroenteritis in humans and other animals. In the city of Campinas in southeast Brazil, giardiasis is endemic, and this pathogen is detected at high concentrations in wastewater effluents, which are potential reservoirs for transmission. The Samambaia wastewater treatment plant (WWTP) in the city of Campinas employs an activated sludge system for sewage treatment and ultraviolet (UV) light for disinfection of effluents. To evaluate this disinfection process with respect to inactivating *G. duodenalis* cysts, two sample types were investigated: (i) effluent without UV disinfection (EFL) and (ii) effluent with UV disinfection (EFL+UV). Nude immunodeficient BALB/c mice were intragastrically inoculated with a mean dose of 14 cysts of *G. duodenalis* recovered from effluent from this WWTP, EFL, or EFL+UV. All animals inoculated with *G. duodenalis* cysts developed the infection, but animals inoculated with UV-exposed cysts released a lower average concentration of cysts in their faeces than animals inoculated with cysts that were not UV disinfected. Trophozoites were also observed in both groups of animals. These findings suggest that *G. duodenalis* cysts exposed to UV light were damaged but were still able to cause infection.

## 1. Introduction

Giardiasis is an intestinal infection caused by the protozoan *Giardia duodenalis,* and it represents the most commonly reported protozoan infection in humans and other animals. Some of the assemblage A and B subtypes of *G. duodenalis* have the potential for zoonotic transmission [1–3]. Cysts are the environmental stage of this organism, and they remain viable for several months under a range of environmental conditions and are extremely resistant to chemical disinfection. The infectious dose for these cysts is low; 10–25 cysts can cause illness [4]. Acquisition of giardiasis occurs through a faecal-oral route, which can be person to person, foodborne, or through contaminated water (drinking water or during recreational activities), with the last being the most significant means of transmission of this infection [5–7]. 

 A large outbreak of giardiasis recorded in 2003 (Boston, MA, USA), exhibited two modes of transmission, which illustrated the capacity of *G. duodenalis* to spread through multiple means of transmission; these two modes included a common outbreak by exposure to contaminated recreational water and a subsequent prolonged propagation through interpersonal transmission between people in the community [4]. This pathogen was responsible for 132 waterborne outbreaks that occurred in North America and Europe and consequently resulted in a public health concern [8–10]. In Norway (2004), 1,500 people were diagnosed with giardiasis, which was described as the largest waterborne outbreak recorded worldwide. The water treatment plant involved in this outbreak used chlorination for water treatment [11, 12], and it is well established that *G. duodenalis* cysts are resistant to the chlorination process [8, 13]. 

Many studies on the removal of this parasite have been performed in wastewater treatment plants (WWTP) using different treatment processes, and removal efficiencies of approximately 80.0 to 98.4% have been reported for *G. duodenalis* cysts [11, 14]. However, cysts have been detected in effluent samples at concentrations of 10^3^ to 10^4^ cysts L^−1^ throughout the world, and little is known about the infectivity of cysts in these samples [15–18]. 

Ultraviolet (UV) disinfection is of growing interest for use in WWTPs because it has been demonstrated that UV radiation is effective against protozoans and does not generate by-products, which does occur with chlorination and ozonation [13, 19, 20]. However, because the biocidal effect of UV light is caused by the absorption of UV photons by nucleic acids (DNA/RNA) in cells, causing damage to the nucleic acids, the characteristics of the liquid to be disinfected (turbidity for example) are considered a significant parameter for disinfection [21, 22].

Research on the effect of UV radiation on cyst inactivation using *in vitro* methods underestimates the loss of infectivity for *G. duodenalis* cysts following UV exposure. For example, *in vitro* excystation is not a reliable assay for assessing infectivity and does not correlate well with animal infectivity assays [23]. Additionally, vital dye viability assays (using DAPI/PI) significantly underestimate cyst inactivation compared with infectivity, which indicates that vital dye viability assays should not be used to define inactivation [24]. 

Infectivity analyses using animal models are the best method for this purpose because they require the parasite to be able to reproduce and complete its entire lifecycle to be considered infectious [23, 24]. 

The BALB/c nude animal model was based on the fact that nude mice are natural athymic mutants that are susceptible to infection at low doses. This susceptibility permitted the use of a smaller number of individuals per group, which is in accord with ethical aims of animal experimentation [25].

The natural, recessive nude mutation, which was initially reported to cause a loss of hair in homozygous mice, results in a rudimentary thymus that causes a reduction in the number of lymphocytes [26, 27]. 

In the present study, we evaluated the infectivity of *G. duodenalis* cysts recovered from effluent from the Samambaia WWTP disinfected with UV light. Evaluation of infectivity was determined by an animal infectivity assay using BALB/c nude mice. In all experiments, animals inoculated with cysts that were not disinfected with UV light released a higher concentration of cysts in their faeces compared to animals inoculated with UV-exposed cysts. Trophozoites were also observed on histological slides containing duodenal samples from animals in both groups. The UV light reactor used in our experiments was operated at an actual scale; the effluent quality was highly variable (a high concentration of solids in suspension and high turbidity), affecting the performance of UV radiation. 

## 2. Materials and Methods

### 2.1. Wastewater Treatment Plant

The Samambaia wastewater treatment plant (WWTP), southeast of Campinas (22°56′00′′ S, 47°00′00′′ W), treats approximately 70 L/s of sewage using an activated sludge system. In the experimental phase, operating at a real scale, an In-Line 250 Liquids Disinfection System reactor using high intensity UV radiation (Germetec UV & GO Technology/http://www.germetec.com.br) was installed in the WWTP to disinfect the effluent using a 25 to 30 mJ cm^−2^ dose (medium pressure UV light). 

### 2.2. Cyst Recovery

This study was conducted in real scale and therefore subject to the variations of the operational conditions of a WWTP. During two years, two litres of effluent disinfected by UV radiation and of effluent without UV disinfection were collected once in two weeks at the Samambaia WWTP and concentrated following cellulose ester membrane filtration (porosity of 3 *μ*m, diameter of 47 mm, Millipore) in accordance with the method by Franco et al. [28]. The samples were eluted from each membrane by alternately scraping, the membranes with a smooth-edged plastic loop and rinsing the membrane with elution solution for 20 minutes. The resulting liquid was centrifuged at 1,050 ×g for 10 minutes, and the concentrated pellet was washed and centrifuged again. The density of* Giardia *cysts was determined with fluorescent monoclonal antibody tests (IFA) (Merifluor kit, Meridian Bioscience, Cincinnati, Ohio) according to the manufacturer's instructions. The samples were then maintained in a 2% potassium dichromate solution. 

### 2.3. Infectivity Assay

Groups of 3-4-week-old female nude BALB/c mice (Pasteur lineage) were purchased from CEMIB (Multidisciplinary Centre for Biological Investigation—UNICAMP) with the assurance that the mice were free from all possible protozoan infections. The animals were divided into the following 4 treatment groups according to the inoculum tested: (i) effluent with UV disinfection: EFL+UV (*n* = 3); (ii) effluent without UV disinfection: EFL (*n* = 3); (iii) effluent filtered through cellulose ester membranes (nominal porosity 3 *μ*m) and thus presumably free of *G. duodenalis* cysts: EFLF (*n* = 2); and (iv) sentinel animals, which were not inoculated: ST (*n* = 1) to check for intercage contamination. To prevent undesirable microbial infections in these animals, prior to inoculation, the samples were sanitised with 1.0% sodium hypochlorite (60 minutes, 4°C), and the bleach was then washed out. All mice, the EFL+UV and EFL groups were intragastrically inoculated with a mean dose of 14 cysts/10 *μ*L. The inoculum was obtained from a pool of cysts recovered from four samples chosen at random from the samples collected over 2 years. The number of cysts in the effluent samples was determined by IFA. Mice were caged individually and maintained in isolation. From day 5 to 15 after infection; faeces from all animals were collected and stored in 2% potassium dichromate and assayed by zinc sulphate flotation (specific gravity = 1.2) to isolate cysts. At day 15 after inoculation, all animals were necropsied. A piece of the small intestine of each mouse was removed and fixed for histological examination, and mucosal scrapings from the duodenum and ileum were stained with Giemsa to detect trophozoites (the whole field was observed). Mice were recorded as *Giardia *infected if cysts or trophozoites were found in faecal specimens, in Giemsa-stained intestinal scrapings, or in histological slides (six slides/mice). 

The protocol used in these experiments was submitted to the Commission of Ethics in Animal Experimentation/CEEA-IB-Unicamp (protocol 909-1) and was approved as being in accordance with the Ethical Principles in Animal Experimentation adopted by the Brazilian College of Animal Experimentation (COBEA).

### 2.4. Genotyping of *Giardia* spp. Cysts

Molecular identification of *Giardia* in the samples was performed by polymerase chain reaction (PCR) of fragments of the *Giardia β-Giardin * gene [29]. Characterisation of the genotype of *Giardia duodenalis* cysts present in the samples was performed by amplification and sequencing of a fragment of the *glutamate dehydrogenase gene* (GDH) [30].

DNA was extracted from cysts using a DNA ZR Fungal/Bacterial DNA Kit (Zymo Research). Two fragments of the *Giardiaβ*-giardin gene were amplified by PCR. The primers GGL639-658 (5′-AAGTGCGTCAACGAGCAGCT-3′) and GGR789-809 (5′-TTAGTGCTTTGTGACCATCGA-3′) generate 171-bp products, and the primers GGL405-433 (5′-CATAACGACGCCATCGCGGCTCTCAGGAA-3′) and GGR592-622 (5′-TTTGTGAGCGCTTCTGTCGTGGCAGCGCTAA-3′) generate 218-bp products. Both were used to confirm the presence of *Giardia* in the samples [29].

The two reactions were performed in a 25 *μ*L volume containing 1x PCR buffer, 2,5 mM MgCl_2_, 0,2 mM each dNTP, 25 pmol of each primer, 1,25 U of Taq polymerase platinum (Invitrogen), and 1 *μ*L of DNA template from cysts isolated from effluent samples. PCR amplification was conducted in a thermocycler (MJ PTC 100-MJ Research INC). The samples were denatured at 94°C for 3 minutes, followed by 40 cycles of 94°C for 1 minute, 60°C for 1 minute, and 72°C for 1 minute. Final elongation was performed at 72°C for 7 minutes. The obtained products were confirmed by 3% agarose gel electrophoresis with ethidium bromide staining. 

A region of the GDH gene was amplified by nested PCR using the primers GDH1 (5′-ATCTTCGAGAGGATGCTTGAG-3′) and GDH4 (5′-AGTACGCGACGCTGGGATACT-3′) in a first round of PCR [30]. The primers GDHF3 (5′-TCCACCCCTCTGTCAACCTTTC 3′) and GDHB5 (5′-AATGTCGCCAGCAGGAACG 3′) were used in a second round of PCR as described by Abe et al. [31].

The two reactions to amplify the GDH gene contained 1x PCR buffer, 2 mM MgCl_2_, 0,2 mM each dNTP, 12,5 pmol of each primer, 0,625 U of Taq polymerase platinum (Invitrogen), and 1 *μ*L of DNA of effluent sample. PCR amplification was conducted in a thermocycler (MJ PTC 100-MJ Research INC). The samples were denatured at 94°C for 3 minutes, followed by 40 cycles of 94°C for 30 seconds, 59°C for 30 seconds and 72°C for 1 minute. Final elongation was performed at 72°C for 7 minutes. The first reaction was performed in a volume of 25 *μ*L, of which 12 *μ*L was used as a template for the second reaction, which was performed in a volume of 100 *μ*L. The obtained products were confirmed by 3% agarose gel electrophoresis with ethidium bromide staining. 

Before sequencing the obtained GDH gene fragment, the products of the second round of PCR were purified using the QIAGEN QIAquick PCR kit. The sequencing reaction was performed using the ABI Prism Big Dye Terminator Cycle Sequencing kit ver. 3.1 (Applied Biosystems), and the samples were sequenced in both directions at least four times using GDHF3 or GDHB5 primers. SeqMan ver. 5.01 (DNASTAR) software was used to edit the sequences. Reference GDH gene sequences from the seven major *G*. *duodenalis* assemblage (A, B, C, D, E, F, and G, GenBank accession numbers L40509.1, L40508.1, U60985.1, U60986.2, AY178740, AY178744, and AY178748.1, resp.) were aligned using Clustal X software [32]. The homologous nucleotide sequence from *Giardia ardeae* (accession number AF069060.2) was used as an out group. Phylogenetic analyses were conducted in the software MEGA ver. 5.05 [33] using neighbour-joining and maximum likelihood algorithms. The model of nucleotide substitution that best fit the data was determined after analysis with jModelTest software [34]. Tamura-Nei93 distance [35] with gamma correction was used and bootstrap phylogeny test was performed in both methods with 10,000 replicates. 

### 2.5. Statistical Analysis

Student's *t*-test with a *P value*, 0.005 was used to analyse the data.

## 3. Results 

**Figure 1:**
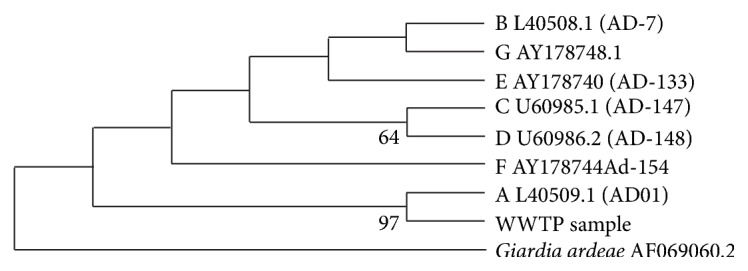
Phylogenetic analysis of wwtp positive sample from *Giardia* glutamate dehydrogenase gene (partial cds). The alignment was generated using Clustal W and analysed using maximum likelihood with the Tamura-Nei 93 model (MEGA v5.05). Trees derived using neighbor joining produced a similar topology. Percentage bootstrap (10000 replicates) is shown beside each node, where >50%. Accession numbers for gdh reference sequences are shown besides the corresponding assemblage.

Amplification of fragments of the *β*-giardin gene and the 220-bp fragment of the GDH gene demonstrated the presence of *G. duodenalis* in the effluent samples from Samambaia WWTP. The sequencing reaction performed for the amplified fragment from the GDH gene using DNA from a positive sample confirmed the presence of *G. duodenalis* assemblage A (Figure 1). The sequence that was obtained has been deposited in GenBank under accession number JN116502.

**Figure 2:**
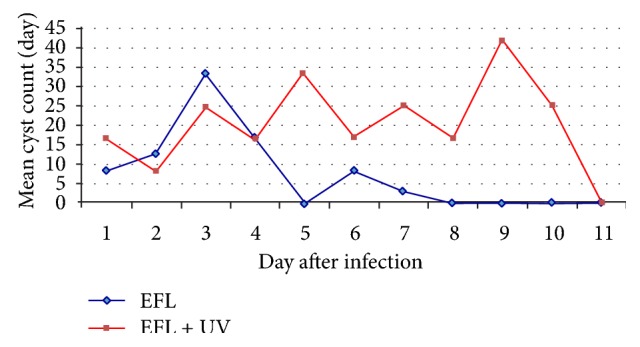
Mean number of *Giardia duodenalis* cysts observed in the faeces of mice from the 5th to 15th day after infection. The control group animals did not eliminate cysts throughout the experiment.

*G. duodenalis* cysts were not detected in the faeces of animals inoculated with effluent filtered through membranes with a porosity of 3 *μ*m (EFLF-negative control), indicating that the filtration process used for this effluent sample was efficient. The sentinel animal (ST) was also negative for cysts in the faeces, indicating that no contamination occurred inside the isolator. The animals in the EFL+UV group eliminated cysts in their faeces from the 5th day to the 11th day after inoculation. The animals in the EFL group eliminated cysts in their faeces beginning on the 5th day after inoculation, and this persisted to the end of the experiment (Figure 2).

With respect to the number of *G. duodenalis* cysts in the faeces per day, we found that the total number was similar between groups EFL+UV and EFL. However, there was a significant statistical difference between the groups (*P* > 0,005). The animals of group EFL eliminated an average of 36.7 cysts/day (varying from 8.3 to 41.6 cysts/day) during the 15 days of the experiment, with the highest number of cysts being eliminated on the 13th day. The animals in the EFL+UV group eliminated an average 16.6 cysts/day (varying from 8.3 to 33.3 cysts/day), with the maximum number of cysts being eliminated on the 11th day.

**Figure 3:**
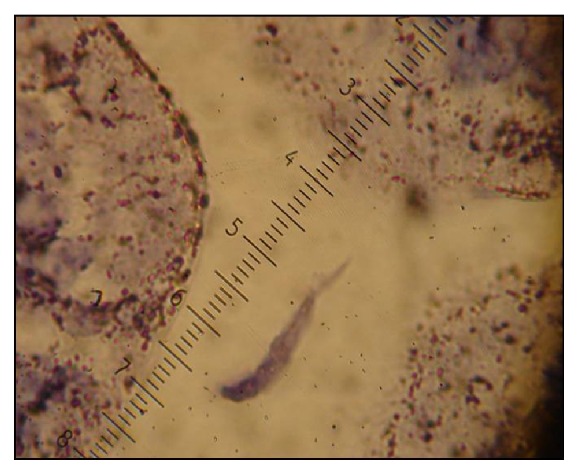
Trophozoite in histological slides from mice inoculated with cysts of *Giardia duodenalis* obtained from treated wastewater and disinfected with UV light.

Neither the intestinal scrapings from the duodenum/ileum nor the enteric histological slides from animals in the EFLF and ST groups were positive for trophozoites. In contrast, the groups inoculated with *G. duodenalis* cysts (EFL+UV and EFL) trophozoites were observed (Table 1 and Figure 3). 

**Figure 4:**
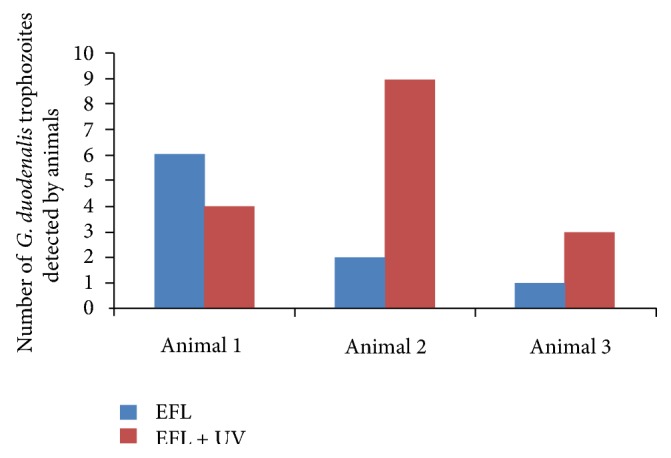
Trophozoite number in histological slides from mice inoculated with cysts of *Giardia duodenalis* obtained from treated wastewater either disinfected or not with UV light.

There was a difference observed with respect to the number of cysts released by the animals that developed the infection and in the number of trophozoites found in the microscopic slides (Figure 4). 

Cysts were also observed in histological slides for samples obtained from animals in the EFL+UV group.

## 4. Discussion

The cysts detected in effluent from Samambaia WWTP and used to infect mice following UV light disinfection were designated as belonging to assemblage A. Assemblage A infects most vertebrates. Additionally, assemblages A and B are the only two assemblages known to infect humans and are thus considered to have zoonotic potential. The effluents from this WWTP after UV light disinfection are discharged into the streams of Pinheiros, which then flow to the Atibaia River. Several cities use the waters of this river as a water supply source, including Campinas, which is the third largest city in São Paulo state. Given that this pathogenic agent has a major impact on public health, these results show the importance of protecting surface water sources from sewage discharges.

Intragastric inoculation of mice with *G. duodenalis* cysts resulted in infection, as assessed by cysts eliminated in their faeces and presence of trophozoites in their small intestines. Our observation of cysts on the 5th day after inoculation is in accordance with kinetics of *Giardia* infection observed in mice and with the capacity of the BALB/c strain to eliminate the infection in a short period of time compared to the other strains [36, 37]. 

Typically, peak cyst release was observed at some point during the first 10 days of infection, followed by a decline in the cyst output and eventual elimination of the parasite at the end of 2 weeks.

The results obtained in this study confirm that acquisition of this infection may occur at low doses. All of the animals in the EFL and EFL+UV groups inoculated with approximately 14 cysts developed an infection, but the infection was associated with an absence of clinical signs and there was a difference in infection intensity between groups. It was observed that the intensity of infection was significantly lower in animals of group EFL+UV than animals of group EFL. 

A lower level of inactivation of the cysts by the UV reactors may be explained by the UV dose delivered to the cysts being lower than necessary, the potential repair of cysts following UV radiation exposure, and the protection of cysts from inactivation due to particle shielding effects [37]. The radiation dose evaluated in this study was 25–30 mJ cm^−2^, and several studies on the inactivation of protozoa by UV radiation have shown inactivation at a UV dose of 20 mJ cm^−2^. Disinfection of *G. duodenalis* cysts with a UV dose of 1 mJ cm^−2^ based on evaluation in an infectivity assay (*Meriones unguiculatus*) was reported by Shin et al. [20], suggesting that the UV radiation dose used by the Samambaia WWTP should have been appropriate for the inactivation of *G. duodenalis* cysts.

It is possible that repair of the cysts may have occurred, but the repair mechanism was not evaluated in this study. Rochelle et al. [38] showed that *Cryptosporidium* spp. protozoa contain genes necessary for repair. However, there is no evidence that UV light-exposed oocysts can be sufficiently repaired to regain their preexposure levels of infectivity. Nevertheless, other components of the cell may be damaged by the UV disinfection performed [8, 13]. 

The effectiveness of UV radiation is directly related to the UV dose absorbed by microorganisms, and the characteristics of the liquid to be disinfected. For instance, turbidity and the concentration of solids in suspension are considered significant parameters affecting the performance of UV radiation for disinfection. There are 3 principal factors involved in this interference: (a) the number and size of particles, which could cause dispersion of radiation and occurrence of shading areas; (b) the nature of particles (organic or inorganic); and (c) the degree of association of microorganisms and particles, which protects the microorganisms from UV radiation [22]. Thus, the properties of wastewater effluent may not be favourable for disinfection of the effluent by exposure to UV light [13, 19]. 

Based on faecal coliform inactivation, some studies indicate that suspended solid concentrations and turbidity are greatly reduced by sand filtration of secondary effluent wastewater samples and that this increases the performance of UV disinfection significantly [39]. In the case of this study, *G. duodenalis* cysts recovered from the effluent of Samambaia WWTP maintain the ability to cause infection. Samambaia WWTP was designed to produce secondary effluent wastewater through biological treatment. The effluent quality observed during this study showed an average turbidity of 13.37 NTU (range 1.75 to 25.00) and average concentration of solids in suspension of 18.00 mg L^−1^ (range 2.00 to 34.00). These parameters correspond to samples analysed in the infectivity assay. Therefore, the UV disinfection employed in this case was not able to inactivate *G. duodenalis* cysts, which was attributed to effluent quality [15]. Li et al. [40]. assessed the infectivity of cysts in wastewater following disinfection with UV radiation and also reported, based on an animal infectivity assay, that this disinfection process was not completely efficient for inactivation of *Giardia* spp. 

Even with their inherent variability, infectivity assays provide a direct measure of the ability of cysts to cause infection and represent the best method for the purpose of evaluating the efficiency of various processes of disinfection in the inactivation of pathogens, such as *G. duodenalis* cysts. However, it is necessary for researchers to obtain standardised protocols to minimise the variables associated with these assays.

The results obtained in this study have great public health relevance, considering that conventional processes of sewage treatment using activated sludge do not result in total removal of protozoan pathogens, and disinfection by light UV is not able to achieve complete inactivation of these pathogens before discharge to the environment (into streams). Individuals infected with a few cysts or trophozoites have the same probability of disseminating the parasite as those receiving high doses [41, 42]. Sewage effluent is an important source of environmental contamination, particularly when it is water supplied for drinking, recreation, or agricultural purposes that is contaminated. The possibility exists that upon drinking this water, individuals infected with a decreased dose of cysts will develop subclinical giardiasis, as shown from the results obtained in this study, in which the animals in our model developed the sub-clinical form of this illness.

Giardiasis is well recognised as causing chronic infections. Thus, *G. duodenalis* cysts present in sewage influent cannot only be derived from symptomatic individuals, but also from asymptomatic persons. It is therefore noteworthy that a lack of clinical symptoms may preclude a search for treatment, and hence, asymptomatic individuals act as an important source of dispersion of *G. duodenalis* cysts.

## Figures and Tables

**Table 1:** Presence of cysts and trophozoites in faeces, scrapings, and histological preparations from the duodenum and ileum of mice inoculated with cysts of *G. duodenalis* obtained from treated wastewater disinfected (or not) with UV light.

Groups	Cysts (faeces)	Cysts (scrapings)	Trophozoites (scrapings)	Trophozoites (histological slides)
Effluent+UV	+(3/3)^a^	+(1/3)	+(1/3)	+(3/3)
Effluent	+(3/3)	−(0/3)^b^	+(2/3)	+(3/3)
Effluent filtrated	−(0/2)	−(0/2)	−(0/2)	−(0/2)
Sentinel	−(0/1)	−(0/1)	−(0/1)	−(0/1)

^a^The presence of cysts in faeces or intestinal trophozoites was found to be infection = positive and,

^b^if not detected = negative.
